# Honey Extracts Exhibit Cytoprotective Properties against UVB-Induced Photodamage in Human Experimental Skin Models

**DOI:** 10.3390/antiox9070566

**Published:** 2020-06-30

**Authors:** Athanasios Karapetsas, Georgia-Persephoni Voulgaridou, Dimitra Iliadi, Ilias Tsochantaridis, Panagiota Michail, Spyridon Kynigopoulos, Maria Lambropoulou, Maria-Ioanna Stavropoulou, Konstantina Stathopoulou, Sofia Karabournioti, Nektarios Aligiannis, Konstantinos Gardikis, Alex Galanis, Mihalis I. Panayiotidis, Aglaia Pappa

**Affiliations:** 1 Department of Molecular Biology & Genetics, Democritus University of Thrace, 68100 Alexandroupolis, Greece; karapetsas_than@yahoo.gr (A.K.); georgiavou_85@hotmail.com (G.-P.V.); dimitrailiadi@gmail.com (D.I.); iliatsoc@gmail.com (I.T.); peny_mich@hotmail.com (P.M.); agalanis@mbg.duth.gr (A.G.); 2 Laboratory of Histology and Embryology, School of Medicine, Faculty of Health Sciences, Democritus University of Thrace, 68100 Alexandroupolis, Greece; spyroskinigopoulos@hotmail.com (S.K.); mlambro@med.duth.gr (M.L.); 3 Department of Pharmacy, Division of Pharmacognosy & Natural Products Chemistry, University of Athens, 15771 Athens, Greece; mstavropoul@yahoo.gr (M.-I.S.); kstatho@pharm.uoa.gr (K.S.); aligiannis@pharm.uoa.gr (N.A.); 4 Attiki-Pittas, Department of Quality Assurance, 15771 Athens, Greece; scar@attiki-pittas.gr; 5 APIVITA SA, Industrial Park, Markopoulo, 19003 Athens, Greece; gardikis-k@apivita.com; 6 Department of Electron Microscopy & Molecular Pathology, The Cyprus Institute of Neurology & Genetics, Nicosia 2371, Cyprus; mihalisp@cing.ac.cy; 7 The Cyprus School of Molecular Medicine, Nicosia 1683, Cyprus

**Keywords:** honey, antiaging, antioxidant, photoprotective, ultraviolet radiation (UV), DNA damage, matrix metalloproteinases (MMPs), HaCaT, 3D reconstituted skin model

## Abstract

In the present study, we aimed to examine the antioxidant, antiaging and photoprotective properties of Greek honey samples of various botanical and geographical origin. Ethyl-acetate extracts were used and the and the total phenolic/flavonoid content and antioxidant capacity were evaluated. Honey extracts were then studied for their cytoprotective properties against UVB-induced photodamage using human immortalized keratinocytes (HaCaT) and/or reconstituted human skin tissue models. Specifically, the cytotoxicity, oxidative status, DNA damage and gene expression levels of specific matrix metalloproteinases (MMPs) were examined. Overall, the treatment of HaCaT cells with honey extracts resulted in lower levels of DNA strand breaks and attenuated the decrease in cell viability following UVB exposure. Additionally, honey extracts significantly decreased the total protein carbonyl content of the irradiated cells, however, they had no significant effect on their total antioxidant status. Finally, the extracts alleviated the UVB-induced up-regulation of MMPs-3, -7 and -9 in a model of reconstituted skin tissue. In conclusion, honey extracts exhibited significant photoprotective and antiaging properties under UVB exposure conditions and thus could be further exploited as promising agents for developing novel and naturally-based, antiaging cosmeceutical products.

## 1. Introduction

Human skin is the largest multifunctional organ of the body, and it plays a pivotal role in temperature regulation, detection of environmental stimuli, and in protecting the body against external biological, chemical and physical stressors [1,2,3]. Serving as a physical barrier, skin is constantly exposed to a wide range of environmental agents such as microbes, irritants, allergens, pollutants and ultraviolet (UV) radiation. Specifically, UV radiation can have detrimental effects on skin homeostasis such as erythema and immunosuppression, and it has also been associated with skin aging and carcinogenesis [4]. 

UV can be subdivided into three main categories according to wavelength: UVC (200–290 nm), UVB (290–320 nm) and UVA (320–400 nm) [5]. While UVC is the most damaging type of UV radiation, it is mainly absorbed by the ozone layer of the atmosphere and thus does not reach the earth’s surface. On the contrary, UVB is only partially absorbed by the ozone layer, therefore, it reaches the skin epidermis, while UVA can penetrate deeper into the skin dermis as it is not absorbed by the atmosphere [6,7].

UV is strongly linked to skin aging through various molecular and cellular mechanisms [8]. Particularly, UVB radiation is the most detrimental radiation for the epidermal basal layer as it directly induces the formation of UV-induced DNA photoproducts with the most prominent ones being cyclobutane pyrimidine dimmers (CPDs) and pyrimidine 6–4 pyrimidines [9]. These photolesions can inhibit DNA replication and transcription and therefore generate permanent mutations, which subsequently lead to cell death, photoaging and photocarcinogenesis [10]. Moreover, UVB irradiation is linked to collagen degradation through inhibition of pro-collagen biosynthesis and up-regulation of matrix metalloproteinases (MMPs) [11]. These are calcium-dependent enzymes that play a key role in tissue remodelling and extracellular matrix degradation [12]. Additionally, UVA and UVB interfere with the skin aging process through the generation of reactive oxygen species (ROS) [13]. Increased ROS levels lead to oxidative stress and cause damages to biomolecules, such as proteins, DNA and lipids [14]. Hence, the development of cosmeceuticals products with photoprotective, antioxidative and antiaging properties is fundamental for the prevention of the hazardous effects of UV exposure.

Since ancient times, honey is well-known for its therapeutic potential [15]. In recent years, there is a tremendous interest in related potent bioactivities, antiseptic and/or health-promoting properties [16]. Honey is a concentrated aqueous solution of inverted sugars, mainly glucose and fructose, which contains a variety of amino and organic acids, vitamins, minerals and antioxidants. It is rich in flavonoids (apigenin, quercetin, galangin, kaempferol, pinocembrin, acacetin), phenolic acids (caffeic acid, gallic acid), caffeic acid phenethyl ester (CAPE) and carotenoids [16]. Different types of honey exhibit wide variations in their composition as a result of the diversity of bee species and their foraging strategies, as well as differences in weather conditions and botanical sources at the collection sites [17,18].

Several studies have indicated the antibacterial [18,19,20,21,22,23,24,25,26], antioxidant [21,22,23,24,26,27,28,29,30], antifungal [18,21], antimicrobial [21,24,25,27,28,30], anti-inflammatory [18,19,21,22,23,24,25,26,27,28,30], antitumor [18,21,22,26,27,28], immunomodulatory [18,19,21,24,25,26,27,28,30] and antiproliferative [21,22,26,27,28] potential of honey. The antioxidant properties of honey are mostly attributed to its organic acids [24] as well as to its phenolic components, which are mainly flavonoid compounds [16,21,22]. Honey could exert its antioxidant activity through scavenging ROS, elevating the intracellular levels of glutathione (GSH), beta-carotene, uric acid and vitamin C, donating hydrogen and chelating metallic ion [23,31]). The phenolic content of honey is also related to its anti-ulcerous, anti-inflammatory, and anti-bacterial properties [24,27,32]. Specifically, certain phenolic ingredients of honey inhibit the nitric oxide synthase, inducible nitric axide synthase (iNOS) and cyclooxygenase-2 (COX-2) activities. Furthermore, the anti-ulcerous properties of honey phenolic compounds are attributed to their ability to enhance the prostaglandin content of the mucosa as well as to suppress acid secretion. Additionally, chrysin, a significant flavonoid component of honey, was found to protect human HaCaT keratinocytes against UVA and UVB-induced DNA damage, ROS generation and apoptosis, as well as inhibit UV-dependent up-regulation of COX-2 in HaCaT cells. Moreover, anticancer and antimetastatic properties have also been reported [26,33].

However, few reports are available on the photoprotective qualities of honey against UV-related photoaging. The goal of this study was to investigate the protective effects of Greek-origin honey extracts against UVB, which causes the most severe damaging effects in the epidermal skin layer [34,35]. Therefore, samples of honey from various botanical origin and geographic regions of Greece were collected and extracted. The extracts were examined initially for their phenolic and flavonoid content and in vitro antioxidant activity. Then, the cytotoxicity, oxidative status, DNA damage and gene expression levels of specific MMPs were examined under UVB exposure conditions in both human immortalized keratinocytes (HaCaT) and reconstituted skin tissues (EpidermTM EPI-200).

## 2. Materials and Methods

### 2.1. Honey Collection, Melissopalynological Analysis, Extraction and Processing

Five honey samples were collected from various regions of Greece on different seasons (Table 1). They were subjected to melisopalinological analysis according to the method of Von der Ohe et al. [36]. Ten grams (10 g) of each honey sample was diluted in 20 mL of distilled water and centrifuged at 3000 rpm for 10 min. The sediment was dried at 40 °C and mounted with Entellan Rapid (Merck, Kenilworth, NJ, USA, 1.07961.0500). Pollen grains (800–1000) were counted and identified in two different slides at 200× magnifications using an OLYMPUS BX 40 light microscope. Phenolic compound extraction and purification steps were performed as described previously [37]. Samples were thoroughly mixed (100 g) with 500 mL dH_2_O until completely fluid. The mixture was mixed and stirred with the non-ionic macroreticular adsorbent resin XAD-4 at RT for 15 h to obtain the phenolic components, which are responsible for the antioxidant activity. The solution was filtered under vacuum and the resin was washed with dH_2_O to remove sugars. The phenolic compounds absorbed in the resin were eluted with methanol. Since significant amount of sugars were still contained in the phenolic extracts, they were concentrated, dissolved in dH_2_O and forwarded to extraction with ethyl acetate (3 × 50 mL). The ethyl acetate extracts were forwarded for evaluation of their phenolic and flavonoid content as well as for their cell-free antioxidant activity. The most promising extracts were further examined for their photoprotective potential.

### 2.2. Assessment of Total Phenolic Content (TPC)

The total phenolic content of honey extracts was calculated through the Folin–Ciocalteu method [38,39]. Particularly, either 25 μL of honey extracts or standard solution of gallic acid (2.5, 5, 10, 12.5, 20, 25, 40, 50, 80, 100 μg/mL) diluted in DMSO were added to 125 μL of 10% Folin-Ciocalteu solution. Subsequently, 100 μL of 7.5% sodium carbonate were added in a 96-well plate. The samples were incubated for 30 min at room temperature in dark. Measurement of the absorbance and estimation of the TPC expressed as mg gallic acid equivalents (GAE) per gram of dry extract were performed as described previously [40].

### 2.3. Assessment of Total Flavonoid Content (TFC)

The aluminium chloride colorimetric assay was used for the evaluation of the TFC of honey extracts [41] and was carried out as described previously [40].

### 2.4. Evaluation of Cell-Free Antioxidant Activity through ABTS (2,2′-Azino-bis(3-Ethylbenzothiazoline-6-Sulfonic Acid) and DPPH (2,2-Diphenyl-1-Picrylhydrazil) Assays

The radical scavenging activity of honey extracts was estimated by the ABTS and the DPPH assays as described previously [40,42,43,44]. Based on the values derived from the percentage inhibition of the radical for each extract, reference curves were plotted for each extract. From these curves (extract concentration—inhibition %) the IC_50_ (μg/mL) values were calculated. IC_50_ (half maximal inhibitory concentration) value corresponds to the sample concentration that can scavenge 50% of the DPPH radical and is inversely proportional to the antioxidant/radical scavenging of the sample. Gallic acid was used as positive control.

### 2.5. Cell Culture

The immortalized human skin keratinocyte (HaCaT) cell line was purchased from the American Type Culture Collection (ATCC, Rockville, USA) and cultured in Dulbecco’s modified Eagle’s medium (DMEM) high glucose, enriched with 10% foetal bovine serum (FBS), 100 U/mL penicillin and 100 μg/mL streptomycin (all were supplied by Biosera, Boussens, France), at 37 °C, 5% *v/v* CO_2_, in a humidified atmosphere. Honey extracts were dissolved in DMSO and then diluted in culture medium. In all treatments, cells (60–70% confluency) were incubated with honey extracts for 2 h, followed by UVB exposure (55 mJ/cm^2^), then treated with honey extracts for 2 h, and finally recovered in complete medium for 24 h. Cell viability was assessed by trypan blue exclusion assay.

### 2.6. Sulforhodamine B (SRB) Assay

The cytotoxicity profile of honey extracts was assessed by the SRB assay as described previously [40]. In brief, 5 × 10^3^ HaCaT cells per well were cultured in 96-well microplates. After 24 h, cells were incubated with increasing concentrations of honey extracts (0–200 μg/mL) for 24 h and then fixed by 50% (*w/v*) ice-cold trichloroacetic acid (TCA) and stained with 0.4% (*w/v*) SRB dye in 1% (*v/v*) acetic acid. Finally, the bound dye was dissolved in 10 mM Tris base and the absorbance was monitored at 570 nm by a multi-plate reader (Tecan, Mannedorf, Switzerland). The percent (%) cell viability was calculated using the formula:

[(Sample OD_570_ − media blank OD_570_)]/[(mean control OD_570_ − media blank OD_570_)] × 100 (1)

The EC_50_ values (effective concentration that induces 50% decrease in cell viability) were calculated by Sigma Plot Software v.10 (Systat, San Jose, CA, USA) via a four-parameter logistic curve. The correlation of ABTS values with TPC honey extract levels was examined by Pearson coefficient correlation with Graph Pad Prism.

### 2.7. Single Cell Gel Electrophoresis Assay/Comet Assay

For comet assay, 3 × 10^5^ HaCaT cells were plated in 60 mm plates, cultured for 24 h and then incubated in the presence or absence of honey extracts (20 μg/mL) diluted in cell culture medium for 2 h. Following incubation, keratinocytes were washed and irradiated by UVB (55 mJ/cm^2^) in PBS (Biosera) using a UV Stratalinker 1800 (Stratagene, La Jolla, CA, USA) or left untreated. Both treated and untreated cells were further incubated with 20 μg/mL honey extracts diluted in culture medium or with normal culture medium for 2 h and then allowed to recover for 24 h in culture medium. Subsequently, cells were collected with trypsinization and comet assay was performed as described previously [40,45].

### 2.8. Determination of Antioxidant Capacity in Cell Lysates

The Cayman’s Antioxidant Assay kit (Cayman Chemical, Ann Arbor, MI, USA) was used for the estimation of the antioxidant capacity of HaCaT cell lysates according to manufacturer’s instructions and as described previously [40]. In brief, 2.5 × 10^6^ HaCaT cells were cultured for 24 h and incubated with or without 20 μg/mL of honey extracts diluted in cell culture medium for 2 h. Then, the HaCaT cells were UVB irradiated (55 mJ/cm^2^) in PBS by using a UV Stratalinker 1800 (Stratagene, La Jolla, CA, USA) or left untreated. The cells were further incubated for 2 h in culture medium with or without honey extracts (20 μg/mL) and then allowed to recover in culture medium for 24 h. Finally, the cells were collected and lysed and processed for the evaluation of the antioxidant capacity, which was expressed as mM Trolox Equivalents.

### 2.9. Assessment of Protein Carbonyl Content

For the assessment of protein oxidation, the Protein Carbonyl Colorimetric Assay kit (Cayman Chemical, Ann Arbor, MI, USA) was used to measure the levels of protein-bound carbonyl groups. This assay is related to the reaction of 2,4-dinitrophenylhydrazine (DNPH) with protein carbonyls resulting in the production and detection of hydrazone at 370 nm. In summary, 2.5 × 10^6^ HaCaT cells were plated in 100 mm plates 24 h prior to the experiment. Then, keratinocytes were cultured in the presence or absence of honey extracts (20 μg/mL) for 2 h, washed with PBS, and irradiated by UVB (55 mJ / cm^2^) or left untreated. The cells were then treated with or without 20 μg/mL of honey extracts for 2 h and recovered for 24 h in culture medium. Finally, the cells were collected and processed for the assessment of protein carbonyl content as previously described [40]. Protein concentration was measured by using bicinchoninic acid (BCA) protein assay kit according to the manufacturer’s instructions.

### 2.10. Human Reconstituted Skin Tissue Model (EpiDerm^TM^ EPI-200)

Epiderm^TM^ EPI-200 (MatTek Inc. Ellicot City MA, USA), supplied as 24-well culture plates inserts with each specific insert plate containing skin tissue, is a human reconstituted skin model. More specifically, it is a normal, human 3D model of epidermal tissue, which contains neonatal-derived human keratinocytes. These cells were isolated, cultured and differentiated in order to form the layers of epidermis. In addition, these reconstituted epidermal tissues are metabolically and mitotic active, imitating the human skin properties [46]. They are cultured in specific designed plates so that the upper surface of tissues (stratum corneum) is exposed to air, whereas the under surface is exposed to the medium. Skin tissues were equilibrated in EPI-100 assay medium at 37 °C for 24 h under normal moisture conditions containing 5% *v/v* CO_2_ and then maintained in the same medium. During the experiment, the Epiderm^TM^ EPI-200 epidermal tissues were cultured in 6-well plates that contained the medium. Thus, stratum corneum of skin tissues was exposed to air, whereas the stratum basale was exposed to EPI-100 assay medium.

### 2.11. Treatment and UVB Irradiation of EpiDerm^TM^ EPI-200

The upper surface of Epiderm^TM^ EPI-200 skin tissues were pre-treated with honey extracts (20 μg/mL diluted in EPI-100 assay medium) for 2 h and then rinsed with 1 × PBS (× 3). Subsequently, epidermal tissues (cultured in 1 × PBS) were irradiated with UVB radiation (55 mJ/cm^2^) and then topically treated with honey extracts for 2 h. Finally, the specific insert plates with reconstituted tissues were cultured in fresh EPI-100 assay medium and collected 24 h post-UVB irradiation for processing with immunohistochemistry and real-time PCR.

### 2.12. Quantitative Real-Time PCR

Total RNA was extracted from skin tissues using the Trizol reagent (Life Technologies, Thermo Fischer Scientific, Waltham, USA) in line with the manufacturer’s instructions. Five microliters (5 μL) of total RNA was reverse-transcribed into cDNA using the Superscript First-Strand Synthesis Kit for Real Time Polymerase Chain Reaction (RT-PCR) (Life Technologies). Real-time PCR experiments were performed on a StepOne PCR system in MicroAmp^®^ Fast Optical 48-Well Reaction Plates (Thermo Fisher Scientific) using the KAPA SYBR^®^ FAST qPCR Kit (Kapa Biosystems, Wilmington, NC, USA). The PCR program included the following steps: 3 min at 95 °C followed by 40 cycles at 95 °C for 15 s and at 60 °C for 1 min. Following PCR, melting curve analysis was performed in order to detect the presence of by-products and/or primer dimmers. For the quantification of transcripts, the ΔΔCt method was used. Particularly, the difference in the expression of a gene equals 2^−ΔΔCt^, where ΔCt equals the difference between the Ct of the test gene and the reporter gene (in that case the *β-actin*) and ΔΔCt equals the difference between ΔCt of each sample and the ΔCt of the control. All the reactions were carried out in triplicates and the sequences of *MMP-1, MMP-3, MMP-7, MMP-9* and *β-actin* primers are shown in Table 2.

### 2.13. Immunohistochemistry (IHC)

Histopathological examination was performed on 4 mm tissue sections after haematoxylin and eosin (H&E) staining as described before [40]. To detect MMP-1, MMP-3, MMP-7 and MMP-9, Epiderm^TM^ EPI-200 skin tissues were collected 24 h-post UVB irradiation, fixed in formalin and embedded in paraffin. Next, the sections (size: 2 μm) were deparaffinized, rehydrated and treated for 5 min with 0.3% H_2_O_2_ in methanol preventing the endogenous peroxidase activity. These sections were subsequently immunostained with the peroxidase method (Envision System, DAKO, Carpinteria, CA, USA) in line with the manufacturer’s instructions. In short, the sections were blocked with protein block serum-free (DAKO) post-antigen reacquisition and endogenous peroxidase blocking. They were incubated with monoclonal or polyclonal antibodies, specifically for MMP-1 (mouse-raised, 1:750 dilution), MMP-3 (rabbit-raised, 1:100 dilution), MMP-9 (mouse-raised, 1:900 dilution) (all were supplied by Acris Herford, Germany) and MMP-7 (rabbit-raised, 1:100 dilution) (Proteintech, Manchester, UK) at 4 °C, overnight. Then, the sections were incubated with the corresponding secondary antibodies for 1 h at RT, the antibody complexes were dyed with 3, 3′-diaminobenzidine (DAB) solution (0.05%), and finally sections were counterstained with Mayer’s haematoxylin. Sections were then mounted and visualized under a Nikon Eclipse 50i microtome. Control samples were incubated without mouse or rabbit immunization serum (negative control) and the results were assessed in a blinded fashion by an expert and independent pathologist according to the percentage of positively-stained cells in the entire section of each sample. Specifically, a semi-quantitively system was applied for scoring the specimens on a scale of 0 to 3: negative (0) for sections with >10% positively stained cells; low (1) for sections with 10–20% stained cells; moderate (2) for 20–50% positively stained cells; and high for >50% stained cells. Intensity was not separately scored.

### 2.14. Statistical Analysis

Sigma Plot Software v.10 as well as GraphPad Prism 5 were used for the creation of graphs and the statistical analysis. The results are expressed as the mean of ±SD of at least three independent experiments, which were carried out in triplicates. Statistical analysis between controls and treatments were performed by ANOVA followed by Tukey’s *t*-test. A *p* ≤ 0.05 was considered as statistically significant. The correlation of ABTS values with TPC honey extract levels was examined by Pearson coefficient correlation with Graph Pad Prism.

## 3. Results

### 3.1. Chemical Characterization, Assessment of Radical Scavenging Activity and Cytotoxicity Profile of Honey Extracts

Honey samples were collected from various geographic regions of Greece (Table 1) and extracted with ethyl-acetate (a total of five extracts) (Table 1). The TPC and TFC values of the honey extracts were then evaluated. As shown in Table 3, honey extracts exhibited versatile TPC and TFC, ranging between 78.1 to 101.3 mg GAE/g of dry extract and 6.7 to 30.3 mg QE/g of dry extract for TPC and TFC, respectively. Furthermore, the radical scavenging activity of honey extracts was evaluated by the cell-free ABTS and DPPH methods. As shown in Table 4, honey extracts exhibited scavenging activity, varying between 22.8–25.8% and 55.5–67% inhibition against ABTS^•+^ and DPPH^•^ radical formation, respectively. The IC_50_ values (regarding DPPH scavenging activity) for all samples were higher than 200 μg/mL, while the IC_50_ for gallic acid (used as positive control) was 4.5 ± 0.08 μg/mL.

**Figure 1:**
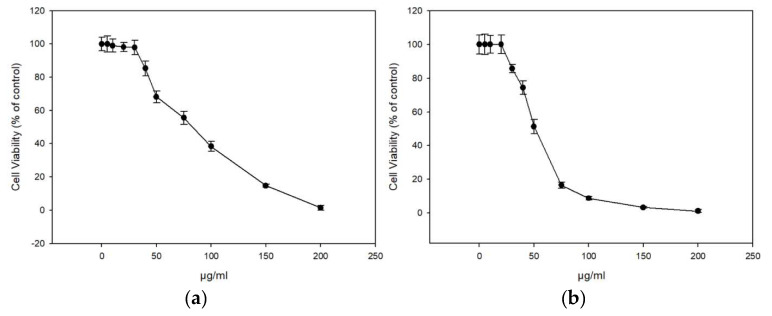
In vitro cytotoxicity assessment of honey extracts in human immortalized epidermal keratinocytes (HaCaT) cells. HaCaT cells were incubated with increasing concentrations of ME16.1 (**a**) and ME20.3 (**b**) honey extracts (0–200 μg/mL) for 24 h. Cell viability was determined by the SRB assay as described in Materials and Methods. The results are shown as ± SD of three independent experiments.

The extracts displayed similar antioxidant profiles based on the ABTS and DDPH assay, and we continued our study with the honey extracts ME16.1 and ME20.3 as these became the first available at sufficient quantities. The cytotoxicity of the extracts were tested in HaCaT cells line using the SRB assay. Cells were treated with increasing concentrations (0–200 μg/mL) of ME16.1 or ME20.3 extracts for 24 h, and cell viability was evaluated as percent of control (untreated cells) viability (Figure 1). The efficient concentrations of the extracts leading to a 50% and 10% decrease in cell viability (EC_50_ and EC_10_, respectively) were also calculated (Table 5). As illustrated in Figure 1, the honey extract ME20.3 exhibited higher cytotoxicity compared to ME16.1 (EC_50_ values were 50.3 ± 2.43 and 91.03 ± 4.23 μg/mL, respectively). However, their EC_10_ values were similar, being 27.14 ± 1.41 μg/mL and 28.87 ± 0.54 μg/mL for ME20.3 and ME16.1, respectively (Table 5). Based on the observation that 20 μg/mL of each extract induced <10% reduction in cell viability, this concentration was further chosen for all subsequent experiments. To assess whether these honey extracts possess UV-screening properties, we analysed the absorption spectrum of the samples ME16.1 and ME20.3. Based on the absorbance spectra, both extracts demonstrated absorption in the UV region (Tables S1 and S2, Figure S1) suggesting a potential photoprotective role.

### 3.2. Honey Extracts Protect HaCaT Cells against UVB-Induced DNA Damage

**Figure 2:**
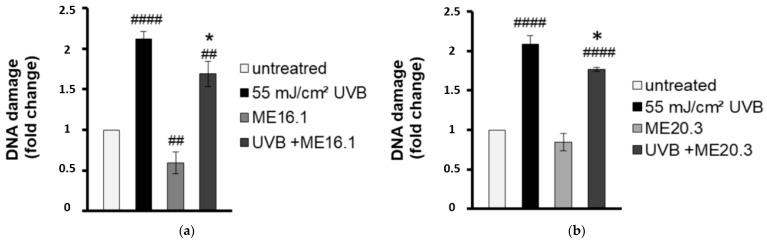
Honey extracts protect HaCaT cells from UVB-induced DNA damage. Cells were pre-incubated with 20 μg/mL of ME16.1 (**a**) or ME20.3 (**b**) for 2 h and then were either UVB-irradiated (55 mJ/cm^2^; treated) or left untreated (control). Treated and control cells were further incubated for 2 h in the presence or absence of honey extracts, left to recover for 24 h and then subjected to single cell gel electrophoresis (comet) assay. Data represent the fold-change of DNA damage in untreated (white), UVB-irradiated (black), honey-treated (light grey) and UVB-irradiated/honey-treated cells (dark grey). Data presented are the mean ± SD of three independent experiments performed in duplicates. ## *p* ≤ 0.01, #### *p* ≤ 0.001, significant differences compared to untreated cells * *p* ≤ 0.05, significant differences between UVB-irradiated and honey-treated/UVB-irradiated cells.

To evaluate the antimutagenic activity of the ME16.1 and ME20.3 honey extracts, we employed the single cell gel electrophoresis assay (comet assay) in order to evaluate the UVB-induced DNA damage in HaCaT cells in their presence or absence. To this end, HaCaT cells were pre-incubated with 20 μg/mL of honey extracts and then were UVB-irradiated (0.2 min, 55 mJ/cm^2^). Following irradiation, cells were further incubated with 20 μg/mL of ME16.1 (Figure 2a) or ME20.3 (Figure 2b) extract for another 2 h and left to recover for 24 h. The comet assay was performed under alkaline conditions to detect both single and double strand DNA breakage. As shown in Figure 2, exposure of HaCaT cells to UVB radiation strongly increased the levels of DNA damage, whereas treatment of HaCaT cells only with the honey extracts had no mutagenic effect. ME16.1 appeared to significantly reduce basal DNA damage levels, but not ME20.3. Importantly, treatment with honey extracts exhibited considerable antimutagenic activity and significantly decreased UVB-induced DNA damage (Figure 2a,b, respectively).

### 3.3. Honey Extracts Protect HaCaT Cells against UVB-Induced Cytotoxicity and Protein Oxidation

**Figure 3:**
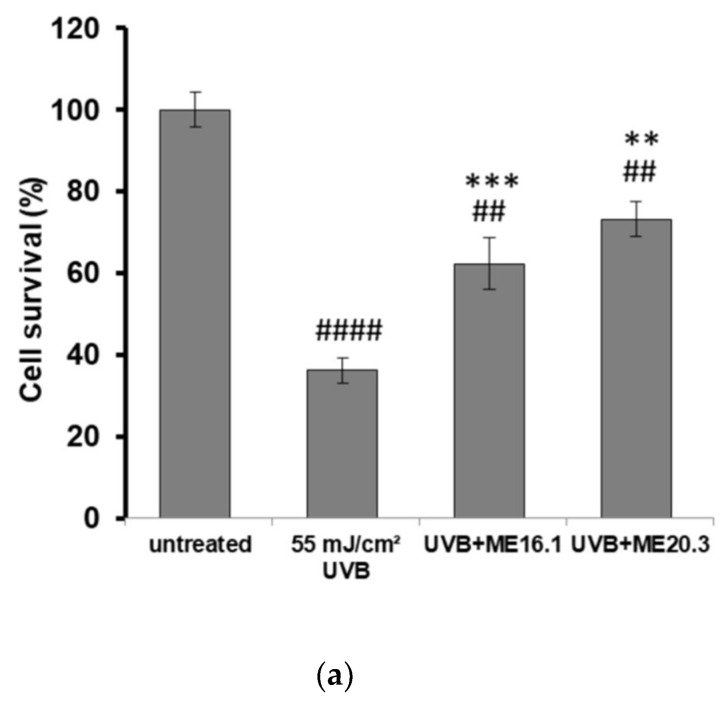

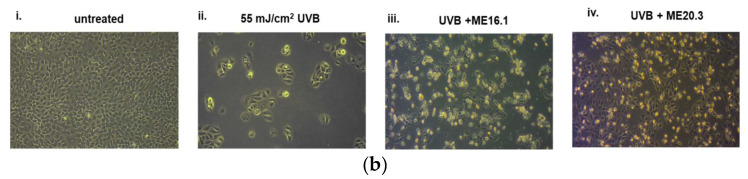
Protection of HaCaT cells by honey extracts against UVB-induced cytotoxicity. Cells were pre-incubated with 20 μg/mL of honey extracts for 2 h, and then were UVB-irradiated (55 mJ/cm^2^) followed by further incubation for 2 h. Following a 24 h recovery period, cell viability was examined by trypan blue exclusion assay and phase contrast microscopy. (**a**) Cell viability determined by the trypan blue exclusion assay. Data presented are the mean ± SD of three independent experiments performed in duplicates. ## *p* ≤ 0.01, #### *p* ≤ 0.001, significant differences compared to untreated cells, ** *p* ≤ 0.01, *** *p* ≤ 0.001, significant differences between UVB-irradiated and honey-treated/UVB-irradiated cells. (**b**) Phase contrast microscopy of non-irradiated (**i**), UVB-irradiated (**ii**) and UVB-irradiated cells treated with ME16.1 (**iii**) and ME20.3 (**iv**). Representative figures of ten random fields for each condition examined in triplicates.

Next, we investigated the potentially protective effects of the honey extracts against UVB-induced cytotoxicity in HaCaT cells, through phase contrast microscopy and trypan blue exclusion assay. As shown in Figure 3, UVB exposure triggered a significant decrease in cell viability (>60%), whereas treatment of HaCaT cells with ME16.1 and ME20.3 honey extracts significantly inhibited UVB-induced cytotoxicity (Figure 3). In the case of treatment with the ME16.1, 62.3 ± 6.44% cell viability was observed, while for ME20.3, 73.23 ± 4.23% cell viability was also observed under conditions of UVB irradiation (Figure 3a). The morphological appearance of HaCaT cells as observed by phase-contrast microscopy under all experimental conditions is illustrated in Figure 3b.

**Figure 4:**
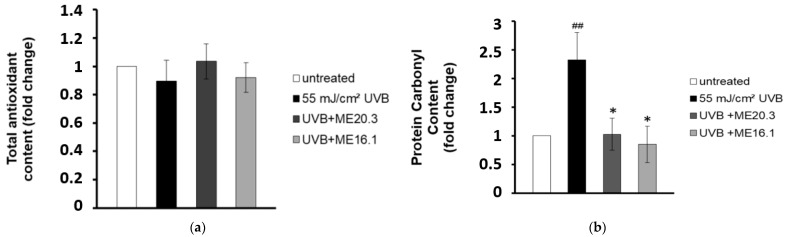
Honey extracts protect HaCaT cells from UVB-induced protein oxidation. HaCaT cells were pre-incubated with 20 μg/mL of honey extracts and then were either exposed to UVB irradiation (55 mJ/cm^2^) or left untreated. Cells were treated for 2 h with honey extracts and then allowed to recover for 24 h. (**a**) Total antioxidant content and activity in HaCaT cell lysates was assessed by the ABTS oxidation assay and expressed as fold change in Trolox equivalents compared to untreated cells. (**b**) Protein oxidation was estimated by measuring the protein carbonyl levels with the DNPH colorimetric assay. The concentration of the protein carbonyls was determined and adjusted to the total protein concentration (expressed as fold-change compared to the untreated cells). Data shown are the mean ±SD of three independent experiments performed in triplicates. ## *p* ≤ 0.01, significant differences compared to untreated cells, * *p* ≤ 0.05, significant differences between UVB-irradiated and honey-treated/UVB-irradiated cells.

In line with the above, we examined whether the protective effects of honey extracts against UVB-induced cytotoxicity were attributed to their antioxidant properties. Therefore, HaCaT cells were pre-incubated with each of the honey extracts for 2 h, exposed to UVB radiation and then incubated again with each honey extract for further 2 h. Cell lysates were subjected to Trolox antioxidant capacity and DNPH protein carbonyl assays. Overall, differences in total antioxidant content and activity between untreated and UVB-irradiated cells (treated with or without the honey extracts) were not significantly different (Figure 4a). On the other hand, UVB irradiation increased the protein carbonyl levels and triggered protein oxidation in HaCaT cells. Remarkably, treatment of the UVB-irradiated HaCaT cells with the honey extracts ME16.1 or ME20.3 substantially decreased the total protein carbonyl levels and thus inhibited protein oxidation (Figure 4b).

### 3.4. Honey Extracts Inhibit UVB-Induced Over-Expression of Matrix Metalloproteinases (MMPs) in a Human Reconstituted Skin Model

**Figure 5:**
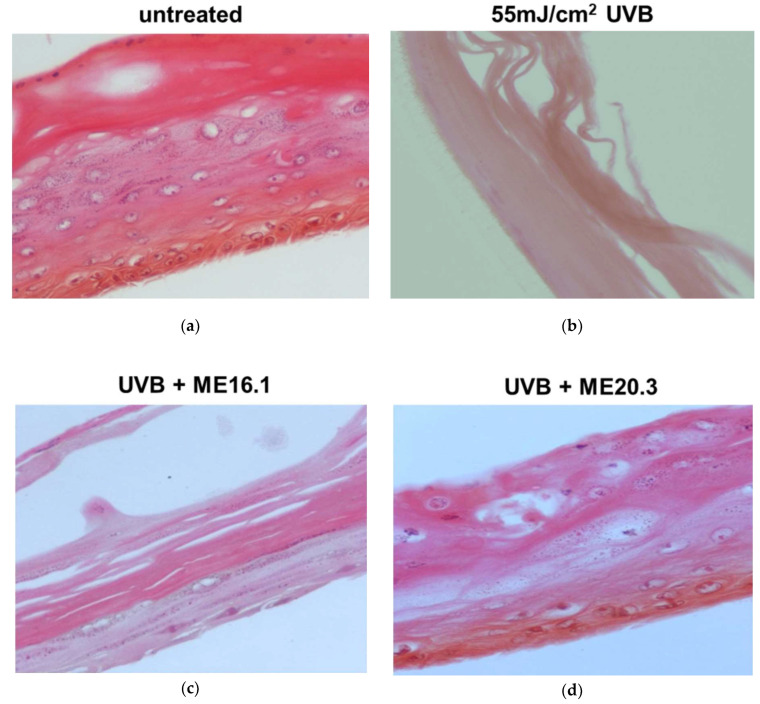
Assessment of the protective effect of honey extracts against UVB-induced skin damage. Epiderm^TM^ EPI-200 reconstituted skin tissues were treated in the apical surface with 20 μg/mL of honey extracts (diluted in assay culture medium) for 2 h, washed with PBS and then exposed to 55 mJ/cm^2^ of UVB irradiation. After UVB exposure, the tissues were incubated with honey extracts for 2 h and then washed with 1 × PBS. After 24 h, the tissues were harvested and sections were taken. Representative images of H&E staining of untreated (**a**), UVB-irradiated (**b**), and pre-treated with ME16.1 (**c**), or ME20.3 (**d**) skin tissues. Magnification ×400.

We further evaluated the protective effects of honey extracts ME16.1 and ME20.3 against UVB-induced photo-aging in the human reconstituted skin model Epiderm^TM^ EPI-200, which simulates human skin. The upper surface of the reconstituted skin tissues was incubated with ME16.1 or ME20.3 for 2 h and then exposed to UVB irradiation (55 mJ/cm^2^). It was then further treated with the honey extracts for an additional 2 h. The medium was changed and then, following 24-h incubation, tissues were harvested and H&E stained in order to observe any UVB-induced skin lesions (Figure 5). Representative pictures of untreated tissues are shown in Figure 5a. UVB irradiation caused severe damage with total necrosis of keratinocytes (Figure 5b). On the contrary, pre-treatment of the UVB-irradiated skin tissues with the ME16.1 resulted in a moderate damage, including a decrease in keratinosomes intercellular oedema and rare sunburn cells (Figure 5c). Similarly, treatment with the ME20.3 extract resulted in moderate damage, including intercellular oedema and few sunburn cells (pyknotic nuclei) in the stratum granulosum layer (Figure 5d).

**Figure 6:**
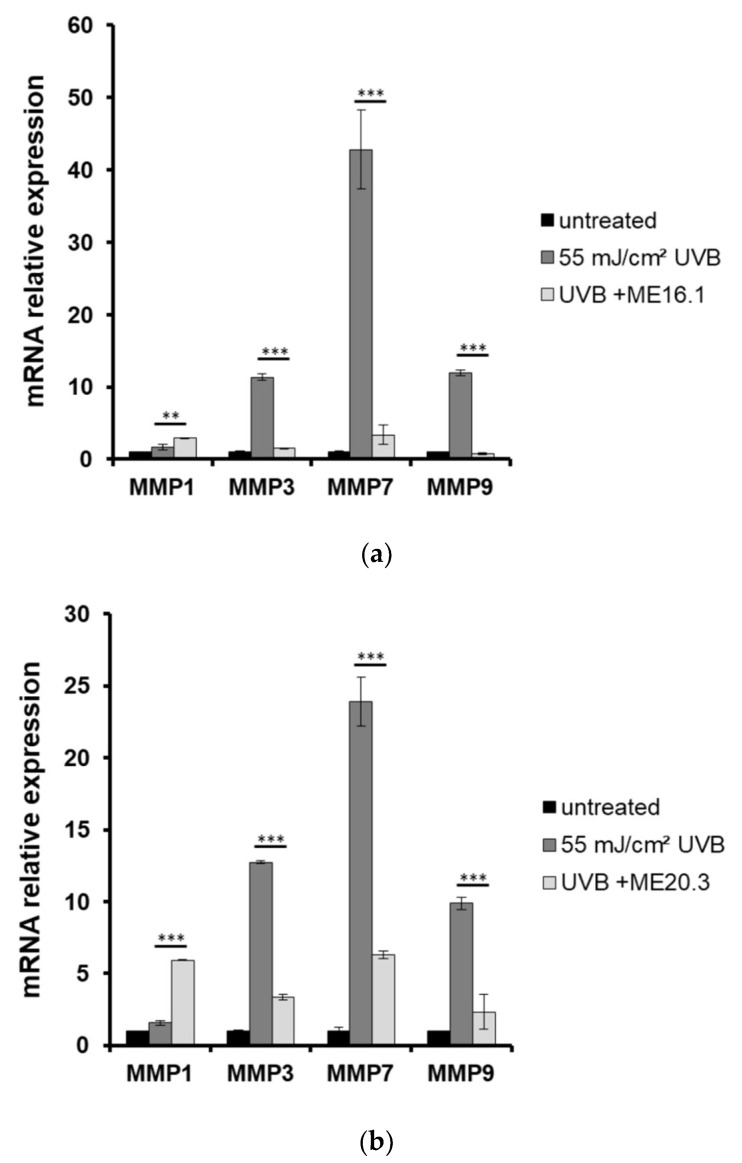
Honey extracts modulate the UVB-induced mRNA expression of MMPs in a 3D reconstituted human skin model (Epiderm^TM^ EPI-200). Tissues were pre-treated with 20 μg/mL of honey extracts ME16.1 (**a**) or ME20.3 (**b**) for 2 h, UVB irradiated (55 mJ/cm^2^) and then treated with honey extracts for 2 h. After 24 h, the tissues were harvested and total RNA was extracted. For the quantification of *MMPs-1*, *-3*, *-7* and *-9* mRNA levels quantitative real-time PCR was performed. The expression levels of all *MMPs* were normalized to those of *β-actin*. Untreated cells served as reference sample. For the relative quantification, the formula RQ = 2^−ΔΔCt^ was used. Representative graphs of three independent experiments. Each reaction was performed in triplicate. ** *p* ≤ 0.01, *** *p* ≤ 0.001, significant differences compared to the UVB-irradiated cells.

Moreover, the expression levels of *MMPs* were estimated by quantitative PCR in skin tissues that had been exposed to UVB and treated with or without pre-treatment with ME16.1 or ME20.3. As shown in Figure 6, UVB irradiation of the reconstituted skin tissues resulted in significant over-expression of all *MMPs* examined. Pre-treatment with ME16.1 caused a significant decrease in the UVB-induced upregulation of *MMPs-3, -7,* and *-9* mRNA levels (Figure 6a). Similar results were documented for the ME20.3 (Figure 6b). Finally, although UVB irradiation did not induce *MMP-1* gene expression levels, significant up-regulation of *MMP-1* was observed in the presence of both honey extracts, being more prominent in the case of the ME20.3.

**Figure 7:**
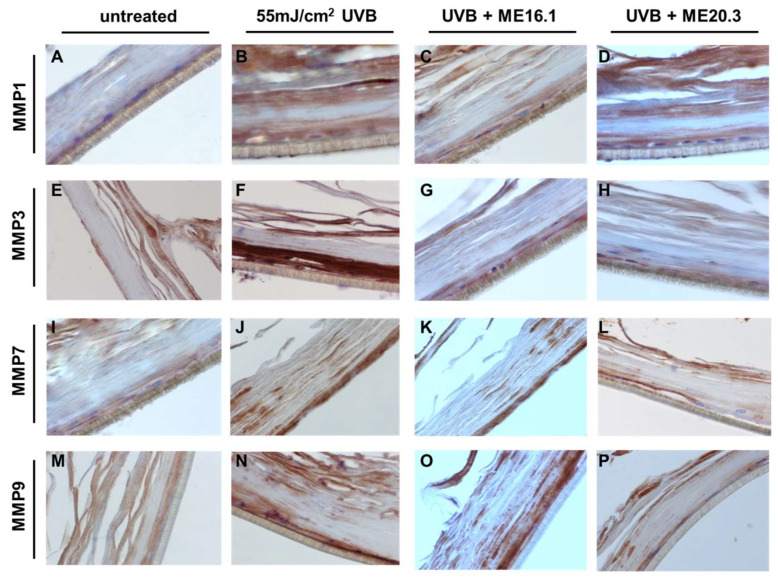
Honey extracts inhibit the UVB-induced expression of MMPs in a 3D reconstituted human skin model (Epiderm^TM^ EPI-200). Tissues were pre-treated with 20 μg/mL of honey extracts ME16.1 or ME20.3 for 2 h, followed by UVB irradiation (55 mJ/cm^2^) and then incubation with honey extracts for 2 h. After 24 h, the tissues were harvested, sections were taken and immunostaining was performed to detect UVB-induced MMP-1 (**A**–**D**), MMP-3 (**E**–**H**), MMP-7 (**I**–**L**) and MMP-9 (**M**–**P**) positive cells. Representative images at 400× magnification of untreated (**A**,**E**,**I**,**M**), UVB-irradiated (**B**,**F**,**J**,**N**), ME16.1 pre-treated/UVB-irradiated (**C**,**G**,**K**,**O**) and ME20.3 pre-treated/UVB-irradiated (**D**,**H**,**L**,**P**) tissues.

To further evaluate the protective effects of honey extracts against the UVB-induced up-regulation of MMPs at the protein level, immunohistochemical analysis was performed. As shown in Figure 7, UVB exposure resulted in over-expression of all the MMPs, and especially those of MMP-3 and MMP-9 (Figure 7B,F,J,N). Treatment with ME20.3 resulted in a significant decrease in the protein levels of all MMPs tested (Figure 7D,H,L,P), compared to UVB-irradiated skin tissues. Similarly, treatment with ME16.1 also decreased the protein expression levels of all MMPs tested (Figure 7C,G,K,O), but to a lesser extent than ME20.3.

## 4. Discussion

Long-term exposure to solar UV radiation causes the accumulation of skin photodamage, resulting in a wide spectrum of skin pathologies, such as inflammation, atrophy, delayed wound healing, carcinogenesis and photoaging [35,47]. The latter is mediated by UV radiation (UVR) through various modes of action, such as the formation of reactive oxygen species (ROS), the induction of DNA damage, the up-regulation of specific MMPs and the inhibition of collagen bio-synthesis [48,49]. Therefore, effective antiaging cosmeceutical agents should ideally possess antioxidant, antimutagenic and antiaging properties. To this end, honey is a natural bee-derived product, widely known for its medicinal and health promoting properties [16]. While, a great number of studies have been focused on its antibacterial, antiviral, anticancer antiproliferative, anti-inflammatory and wound healing capacities [21,27,28,50], its potential as an antiaging agent has not been yet clarified. Therefore, we were prompted to investigate its antioxidant, antimutagenic and antiaging properties under experimental conditions of UVB exposure.

In our study, honey samples were collected from various botanical origins and geographical regions of Greece and extracts were produced. The total phenolic and flavonoid content as well as their in vitro antioxidant activity were initially determined. Two selected samples were further processed and characterized for their antioxidant and photoprotective properties under UVB exposure conditions. Our results indicated that the honey extracts exhibited strong antioxidant and antimutagenic capacity and protected HaCaT cells against the cytotoxic and oxidative effects of UVB irradiation. Furthermore, honey extracts protected against UVB-induced severe photodamage and sufficiently attenuated the UVB-induced MMP up-regulation using a reconstituted human skin tissue model.

The in vitro antioxidant capacity of the extracts was initially studied by employing cell-free well-established methodologies, such as ABTS and DPPH assays. Our results showed that all honey extracts exhibited significant antioxidant activity that may be related with their phenolic and flavonoid content. This finding is in accordance with previous studies demonstrating the antioxidant properties of honey extracts [51,52,53,54,55,56,57]. Interestingly, a strong correlation between the TPC and the antioxidant capacity of honey has been reported by a great number of studies [58,59,60]. However, in our study, all samples, exhibited relatively similar antioxidant capacity, ranging from 18.2% to 25.8% and 51% to 67% inhibition for ABTS and DPPH, respectively. A significant correlation was found between the TPC of the extracts and the ABTS free radical activity (Pearson correlation of 0.61705). On the contrary, no significant correlation was monitored between the TFC and the antioxidant capacity of the extracts, probably because of their low concentration. Quite interestingly, the ME16.1 and ME20.3 extracts demonstrated significant antioxidant activity in HaCaT cells under UVB exposure conditions. Although monitoring the total cellular antioxidant activity did not prove as sensitive and did not reveal any differences between experimental conditions in the absence or presence of the honey extracts, monitoring the cellular protein oxidation levels indicated significant differences. UVB irradiation increased the protein carbonyl levels and triggered protein oxidation in HaCaT cells, and treatment with the honey extracts ME16.1 or ME20.3 substantially alleviated these effects, suggesting strong antioxidant activity of the honey extracts.

The main class of polyphenols in honey are flavonoids and phenolic acids. Flavonoids are mostly recognized as aglycones and not in their glycosylated forms largely due to the presence of glucosidase activity in the salivary glands of bees responsible for the hydrolysis of flavonoid glycosides and the release of the aglycon forms. Phenolic aglycons are more readily absorbed by the gut barrier by passive diffusion and therefore have increased bioavailability [61]. The bioactive flavonoid and phenolic compounds found in different honey types have been recently reviewed [62]. It appears that individual bioactive compounds are similar across different types of honey, but vary in their relative concentrations. Several studies have evaluated the antioxidant properties of honey in vitro and in vivo. The specific honey variety manuka, via its bioactive compounds such as methylglyoxal, decreased intracellular ROS levels, protein and lipid oxidation damage and apoptosis and improved mitochondrial functionality and antioxidant enzyme activities by activating the AMPK-Nrf2 pathway in human dermal fibroblasts [63]. Improved antioxidant status was observed in HepG2 cells treated with bee honey, while an increase in antioxidant enzyme expression and activities has been proved in human diploid fibroblasts treated with monofloral gelam honey [64]. Different types of honey have proved to prevent oxidative damage by reducing ROS levels, inhibiting lipid peroxidation and DNA damage levels and increasing antioxidant enzyme activities in several in vivo models [65,66,67,68].

In our study the honey extracts ME16.1 and ME20.3 effectively protected cells from UVB-induced DNA damage. Our results are consistent with previous findings demonstrating an antimutagenic effect of honey against various genotoxic agents (e.g., UV, H_2_O_2_, pesticides) [69,70,71,72,73]. For instance, Ahmad et al. reported that treatment with tualang honey significantly inhibited UVB-induced formation of CPD and 8-oxodG DNA adducts in the murine epidermal keratinocyte cell line PAM212 [73]. Similarly, Cheng et al. showed that treatment with whole honey resulted in reduced H_2_O_2_-induced DNA damage in mice lymphocytes [69]. In an experimental model of human hepatoma (HepG2) cells, honey samples of different floral origin protected against DNA strand breaks induced by several dietary mutagens, such as benzo(a)pyrene (BaP), 2-amino-1-methyl-6-phenylimidazol (4, 5-b)pyridine (PhIP), N-nitrosopyrrolidine (NPYR), but not N-nitrosodimethylalmine (NMDA). This protective effect was in part attributed to the phenolic content of the honey samples [74]. The post-treatment of human whole blood samples from healthy volunteers with manuka honey resulted in significant protection of whole blood cells from oxidative damage induced by hydrogen peroxide monitored in vitro by comet assay [75]. To the extent of our knowledge, this is the first time that the protective effect of honey against UV genotoxicity has been demonstrated in human keratinocytes.

Additionally, the honey extracts protected HaCaT cells against the cytotoxic, oxidative, and photodamaging effects of UVB. The treatment of HaCaT cells with the honey extracts attenuated the decrease in cell viability following UVB exposure and significantly decreased the oxidative protein damage within the cells, as indicated by decreased total protein carbonyl content following UVB irradiation. The Epiderm^TM^ EPI-200 reconstituted human skin model was utilized to further confirm the photoprotective properties of honey extracts against UVB irradiation. One of the various mechanisms through which UV radiation is related with photoaging is through matrix metalloproteinases (MMPs) up-regulation. MMPs induce alterations of the extracellular matrix (ECM) by degrading proteoglycan, elastin, fibronectin, and collagen, leading to wrinkle formation [76]. Interestingly, the examined honey extracts suppressed the UVB-induced up-regulation of MMPs -3, -7 and -9 both at mRNA and protein level in the reconstituted human skin model. Intriguingly, UVB radiation could marginally induce the expression of *MMP1* at mRNA level, while treatment with the honey extracts could further augment the expression of MMP1. However, this increase was not recapitulated at protein level, as shown by IHC. The same model has been previously used to study the cytoprotective properties of propolis extracts against photodamage [40]. Only a few other reports have investigated the antioxidant, cytoprotective and antiaging effect of honey in skin after UV radiation exposure. For instance, similarly with our data, Majtan et al. demonstrated that honey extracts substantially inhibited the TNF-α-induced up-regulation of MMP-9 in HaCaT cells in a dose-dependent manner [77]. Similarly, Moskwa et al. reported that treatment with honey samples of Polish origin significantly down-regulated MMP-2 and MMP-9 in the human glioblastoma U87MG cell line [78].

To summarize, the results of the present study demonstrate that the tested honey extracts confers protection against UVB-induced skin cellular damage by attenuating UVB-induced toxicity, reducing the cellular and DNA damage levels and modulating the expression levels of several MMPs. These findings suggest that honey could be considered a promising agent for the development of naturally based cosmetic products. Future studies are required to shed more light into elucidating the molecular mechanism(s) underlying the cytoprotective properties of honey under UV exposure conditions as well as to characterize its active components responsible for such specific mode(s) of action.

## Figures and Tables

**Table 1:** Geographic origin and season of harvest of honey samples used in the study.

Honey Extract	Pollen Analysis	Origin	Year of Harvest
ME1.3	*Compositae* 44%	Chania, Crete	2012
	*Thymus capitatus* 21%		
	*Eucalyptus* sp. 11%		
	*Centaurea* sp. 8%		
	*Trifollium* sp. 7%		
	Liliaceae 4%		
	*Erica* sp. 2%		
	*Umbeliferae* 1%		
	Nectarless: *Olea europaea*, *Cistus* sp., *Helianthemum* sp.,		
ME5.3	*Polygonum aviculare*. 38%	Vytina, Peloponnese	2012
	*Rhamnus* sp. 19%		
	*Quercus ilex* 19%		
	*Brassica* sp. 19%		
	*Compositae* 3%		
ME11.2	*Abies* sp. 27%	Feneos, Peloponnese	2014
	*Rosaceae* 27%		
	*Quercus ilex* 18%		
	*Trifollium* sp. 9%		
	*Arbutus* sp. 4.5%		
	*Umbeliferae* 4.5%		
	*Castanea sativa* 4.5%		
	*Ramnus* sp. 4.5%		
	Nectarless: *Cistus* sp., *Olea europaea*		
ME16.1	*Centaurea* sp. 33%	Chania, Crete	2014
	*Compositae* 16%		
	*Ceratonia siliqua* 11%		
	*Quercus ilex* 11%		
	*Lonicera* sp. 11%		
	*Citrus* sp. 11%		
	*Rubus* sp. 5.5%		
	Nectarless: *Cistus* sp., *Convolvulus* sp., *Olea europaea*		
ME20.3	*Trifollium* sp. 3%	Mt. Olympus National Park	2014
	*Carduus* sp. 4%		
	*Mentha* sp. 4%		
	*Abies* sp. 9%		
	*Thymus* sp. 6%		
	Nectarless: *Quercus* sp., *Cistus* sp., *Olea europaea*, *Hypericum* sp., *Rosaceae*		

(sp.: species).

**Table 2:** The primers used for real-time PCR.

Gene	Forward Primer (5′ → 3′)	Reverse Primer (5′ → 3′)
*MMP-1*	CCTCGCTGGGAGCAAACA	TTGGCAAATCTGGCGTGTAA
*MMP-3*	GAGGCATCCACACCCTAGGTT	ATCAGAAATGGCTGCATCGAT
*MMP-7*	CTGCATTTCAGGAAAGTTGTATGG	AGCTCCTCGCGCAAAGC
*MMP-9*	GGACGATGCCTGCAACGT	CAAATACAGCTGGTTCCCAATCT
*β-actin*	GCGCGGCTACAGCTTCA	CTTAATGTCACGCACGATTTCC

**Table 3:** Total phenolic content (TPC) and total flavonoid content (TFC) of honey extracts (C = 0.1 mg/mL).

Honey Extract	TPC mg GAE/g Extract	TFC mg QE/g Extract
ME1.3	101.30 ± 11.20	30.3 ± 4.32
ME5.3	84.60 ± 9.10	9.6 ± 1.23
ME11.2	85.30 ± 6.00	6.7 ± 2.88
ME16.1	78.10 ± 12.50	8.4 ± 0.75
ME20.3	94.40 ± 9.80	9.1 ± 0.88

TPC and TFC values are expressed as mg gallic acid and quercetin equivalents, respectively (GAE and QE), per gram of dry extract.

**Table 4:** ABTS and DPPH free radical activity of honey extracts examined in the present study.

Honey Extract	ABTS Inhibition (%) C = 16.67 mg/mL	DPPH Inhibition (%) C = 0.25 mg/mL
ME1.3	22.90 ± 1.32	55.50 ± 2.33
ME5.3	23.95 ± 2.28	56.10 ±1.25
ME11.2	22.80 ± 3.29	67.00 ± 4.88
ME16.1	18.20 ± 4.55	51.00 ± 5.20
ME20.3	25.80 ± 3.22	61.00 ± 3.89

(C: Concentration).

**Table 5:** The EC_50_ and EC_10_ value of honey extracts ME20.3 and ME16.1.

Honey Extract	EC_50_ (μg/mL) ^1^	EC_10_ (μg/mL) ^1^
ME16.1	91.03 ± 4.23	28.87 ± 1.41
ME20.3	50.3 ± 2.43	27.14 ± 0.54

^1^ Results are presented as the mean ± SD of three independent experiments.

## Supplementary Materials

The following are available online at https://www.mdpi.com/2076-3921/9/7/566/s1, Figure S1: Absorption spectrum (190–800 nm) of honey extracts (ME16.1 and ME20.3) at 20 μg/mL, Table S1: Absorption spectrum of ME16.1 sample in different concentrations (μg/mL), Table S2: Absorption spectrum of ME20.3 sample in different concentrations (μg/mL).
