# Supplementary material for: Honey Extracts Exhibit Cytoprotective Properties against UVB-Induced Photodamage in Human Experimental Skin Models
Source: Antioxidants (Basel). 2020 Jun 30;9(7):566. doi: 10.3390/antiox9070566 (PMC7402120; doi:10.3390/antiox9070566)
Supplement: Supplementary file 1 [file antioxidants-09-00566-s001.pdf]

Absorbance measurement of honey extracts ME16.1 and ME20.3

In brief, honey extracts were diluted in dH_2O to final concentrations 20, 2, and 0.2 µg/ml)(Table S1, S2). Absorption spectrum (190 – 800 nm) of the honey samples was assessed by scanning the absorbance of the solutions in a spectrophotometer (Libra S22, Biochrom Ltd, Cambridge, UK). The absorption spectrum of the honey extracts ME16.1 and ME20.3 at concentration 20µg/ml (working concentration in the study) is illustrated in Figure S1.

Table S1. Absorption spectrum of ME16.1 sample in different concentrations (µg/ml)

	ME16.1		
Wavelength (nm)	20µg/ml	2µg/ml	0.2µg/ml
	Absorbance	Absorbance	Absorbance
190	2.591	2.283	2.332
200	2.954	2.602	2.653
210	>+3	2.758	2.82
220	2.99	2.58	2.694
230	1.006	0.342	0.453
240	0.586	0.073	0.126
250	0.469	0.053	0.102
260	0.457	0.051	0.093
270	0.511	0.055	0.092
280	0.576	0.061	0.089
290	0.637	0.066	0.086
300	0.608	0.063	0.08
310	0.55	0.056	0.074
320	0.506	0.051	0.069
330	0.461	0.048	0.067
340	0.401	0.041	0.063
350	0.334	0.034	0.06
360	0.276	0.028	0.057
370	0.228	0.024	0.054
380	0.189	0.021	0.052
390	0.153	0.017	0.05
400	0.126	0.015	0.048
410	0.106	0.013	0.047
420	0.093	0.012	0.045
430	0.085	0.012	0.045
440	0.079	0.012	0.044
450	0.075	0.011	0.043
460	0.071	0.011	0.042
470	0.069	0.011	0.041
480	0.067	0.011	0.041
490	0.064	0.011	0.04
500	0.062	0.011	0.039
510	0.06	0.01	0.038
520	0.059	0.01	0.038
530	0.058	0.01	0.037

540	0.056	0.01	0.036
550	0.055	0.01	0.036
560	0.054	0.01	0.035
570	0.053	0.01	0.035
580	0.052	0.01	0.034
590	0.051	0.01	0.034
600	0.05	0.01	0.033
610	0.049	0.01	0.032
620	0.048	0.01	0.031
630	0.047	0.01	0.031
640	0.046	0.01	0.031
650	0.046	0.01	0.031
660	0.045	0.01	0.031
670	0.044	0.009	0.031
680	0.043	0.009	0.031
690	0.043	0.009	0.031
700	0.042	0.009	0.031
710	0.042	0.009	0.029
720	0.041	0.009	0.028
730	0.04	0.009	0.028
740	0.039	0.009	0.027
750	0.039	0.009	0.027
760	0.038	0.009	0.027
770	0.038	0.009	0.027
780	0.037	0.009	0.027
790	0.037	0.009	0.027
800	0.036	0.009	0.027

Table S2. Absorption spectrum of ME20.3 sample in different concentrations (μg/ml)

	ME20.3		
Wavelength (nm)	20μg/ml	2μg/ml	0.2μg/ml
	Absorbance	Absorbance	Absorbance
190	2.643	2.359	2.306
200	>+3	2.69	2.634
210	>+3	2.854	2.782
220	>+3	2.556	2.229
230	2.173	0.48	0.321
240	0.946	0.217	0.121
250	0.842	0.194	0.102
260	0.792	0.176	0.093
270	0.719	0.159	0.09
280	0.658	0.148	0.087
290	0.563	0.128	0.08
300	0.433	0.1	0.074
310	0.315	0.077	0.067
320	0.243	0.062	0.064
330	0.193	0.052	0.06
340	0.144	0.042	0.057

350	0.109	0.035	0.054
360	0.083	0.03	0.052
370	0.066	0.026	0.051
380	0.052	0.024	0.049
390	0.04	0.021	0.048
400	0.031	0.019	0.047
410	0.024	0.017	0.047
420	0.02	0.016	0.046
430	0.018	0.015	0.045
440	0.015	0.014	0.044
450	0.013	0.013	0.044
460	0.011	0.013	0.043
470	0.01	0.013	0.042
480	0.008	0.012	0.042
490	0.006	0.01	0.04
500	0.006	0.01	0.04
510	0.005	0.1	0.039
520	0.005	0.009	0.037
530	0.004	0.009	0.037
540	0.004	0.009	0.036
550	0.004	0.009	0.036
560	0.004	0.009	0.036
570	0.004	0.009	0.036
580	0.004	0.009	0.036
590	0.004	0.009	0.036
600	0.003	0.009	0.036
610	0.003	0.009	0.036
620	0.003	0.009	0.035
630	0.003	0.008	0.034
640	0.003	0.008	0.034
650	0.003	0.008	0.033
660	0.002	0.008	0.033
670	0.002	0.008	0.033
680	0.002	0.008	0.033
690	0.002	0.008	0.032
700	0.002	0.008	0.032
710	0.002	0.008	0.032
720	0.002	0.008	0.032
730	0.002	0.007	0.032
740	0.002	0.007	0.032
750	0.002	0.007	0.032
760	0.002	0.007	0.031
770	0.002	0.007	0.03
780	0.002	0.007	0.029
790	0.002	0.006	0.029
800	0.002	0.006	0.029

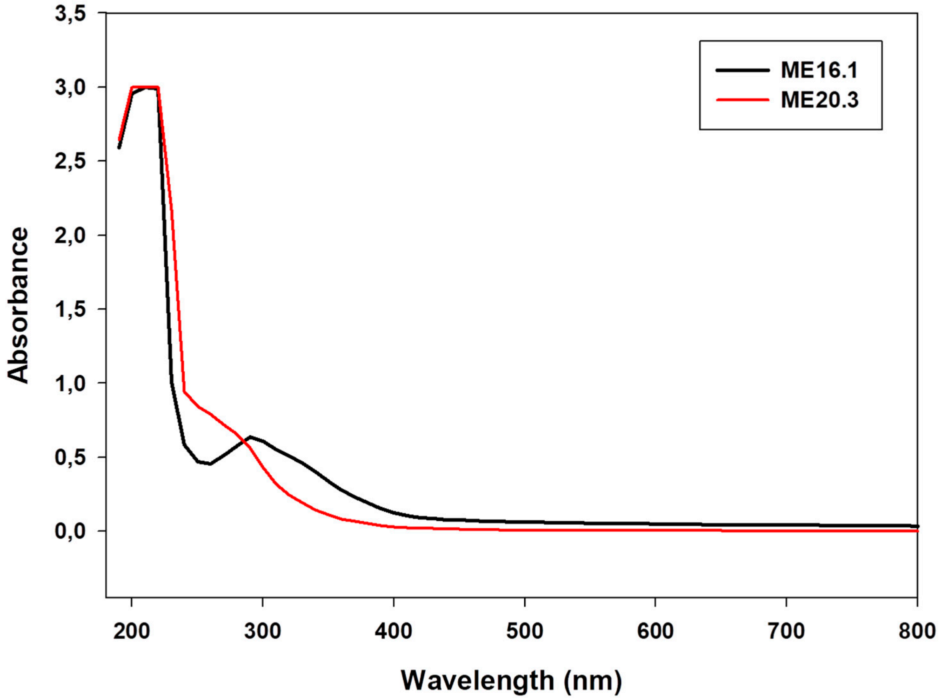

Figure S1. Absorption spectrum (190 – 800 nm) of honey extracts (ME16.1 and ME20.3) at 20μg/ml.
